# The Quality of Clinical Practice Guidelines and Consensuses on the Management of Primary Aldosteronism: A Critical Appraisal

**DOI:** 10.3389/fmed.2020.00136

**Published:** 2020-05-05

**Authors:** Zhe Meng, Liang Zhou, Zhe Dai, Chang Xu, Guofeng Qian, Mou Peng, Yuchun Zhu, Joey S. W. Kwong, Xinghuan Wang

**Affiliations:** ^1^Department of Urology, Zhongnan Hospital of Wuhan University, Wuhan University, Wuhan, China; ^2^Department of Adrenal Hypertension, Zhongnan Hospital of Wuhan University, Wuhan University, Wuhan, China; ^3^Department of Urology, West China Hospital, Sichuan University, Chengdu, China; ^4^Department of Endocrinology, Zhongnan Hospital of Wuhan University, Wuhan University, Wuhan, China; ^5^Chinese Evidence Based Medicine Center, West China Hospital, Sichuan University, Chengdu, China; ^6^Department of Urology, The First Hospital of Zhejiang University, Zhejiang University, Hangzhou, China; ^7^Department of Urology, The Second Hospital of Xiangya, Zhongnan University, Hangzhou, China; ^8^JC School of Public Health and Primary Care, Faculty of Medicine, The Chinese University of Hong Kong, Hong Kong, China

**Keywords:** primary aldosteronism, clinical practice guideline, quality assessment, AGREE II, systematic review

## Abstract

**Background:** Several guidelines and expert consensuses have been developed for management of primary aldosteronism (PA). It is important to understand the detailed recommendations and quality of these guidelines to help physicians make informed and reliable decision.

**Methods:** PubMed, EMBASE, and three websites were searched for practice guidelines or consensuses of PA from inception to January 24, 2019. We summarized the major recommendations on the management of PA from these guidelines and consensuses. The Appraisal of Guidelines for Research and Evaluation II was used to assess quality of the included guidelines and consensuses.

**Results:** We identified three clinical practice guidelines and three consensus statements. Most of the recommendations on the diagnosis and treatment of PA from these guidelines and consensuses were consistent. Some minor conflicts were recorded for patient's screen and confirmation test. All included guideline documents have a good quality (score, >70%) on the scope and purpose (mean score, 81.02%) and clarity of presentation of the recommendations (mean score, 86.88%). However, the reporting for the stakeholder involvement (mean score, 54.32%) and applicability (mean score, 47.92%) were insufficient. There was an insufficient rigorousness in most of the guideline documents (mean score, 45.56%) on the development process. The Endocrine Society practice guideline 2016 ranked highest in quality (score, 81.13%).

**Conclusions:** Existing guideline documents provided valuable recommendations on the management of PA, but further efforts are needed to improve the methodological quality. The Endocrine Society practice guideline 2016 was recommended for use.

## Introduction

Primary aldosteronism (PA) is a group of disorders caused by the autonomous excessive production aldosterone which escapes regulation from angiotensin or plasma potassium concentrations (1). Mass secreting of aldosterone would lead to high levels of potassium in urinary excretion; therefore, PA patients generally had a hypokalemia, severe resistant hypertension, and metabolic alkalosis (2). Patients who suffer from PA may have a higher risk of cardiovascular and cerebrovascular events than those with essential hypertension (3–5). But this excess risk may be mitigated by proper treatment, for example, adrenalectomy for unilateral aldosterone-producing adenomas (6). As a result, a proper management on PA patients is important for the prognosis (7).

Clinical practice guidelines are developed to provide implemental basis for physicians and/or patients for the entire spectrum of clinical decision-making process, from prevention, screening, diagnosis, treatment, to rehabilitation, as an effort to improve the healthcare (8). The potential benefits to the healthcare providers and receivers largely depend on the quality of the guideline itself. Trustworthy guidelines are systematically developed based on reliable evidence, patient-oriented recommendation, and informative disclosure (9).

During the past decades, an increasing number of clinical practice guidelines and consensuses have been developed for the management PA. For example, the Endocrine Society Clinical Practice Guideline, the Chinese Endocrine Society consensus, and the Japanese Endocrine Society guideline (10–12). These guidelines and consensuses form a strong basis of evidence-based recommendations for PA physicians. Some of the recommendations may differ across guidelines. For example, the international Endocrine Society recommended that hypertensive patients with sustained blood pressure (>150/100 mm Hg) should be screened for case detection (10), whereas the Chinese Endocrine Society recommended that patients with sustained blood pressure of 160/100 mm Hg or greater should be screened for case detection (11). Understanding the major discrepancies and the quality of these guidelines and consensuses may be helpful for physicians in clinical practice.

In order to help physicians to make informed and reliable decisions, in this article, we studied the major recommendations and potential discrepancies of current PA guidelines and consensuses; we also conducted a critical appraisal of their quality.

## Methods

### Eligible Criteria, Literature Search, and Screen

We considered both expert consensus and clinical practice guidelines for the management of PA. The definition of expert consensus and clinical practice guideline is available elsewhere (13). In brief, a guideline generally is developed based on existing evidence, whereas consensus may largely rely on the expert experiences. We did not include consensus or guidelines for which the primary objective was outside the scope of PA management. For example, some guidelines for the management of hypertension also contain a small part of recommendation for resistant hypertension caused by PA, which were not considered in current article. In addition, for one guideline that was updated, the latest version would be included for assessment [e.g., the Endocrine Society Clinical Practice Guideline (10)].

PubMed and EMBASE were searched for guidelines or consensus of PA from inception to January 24, 2019. We also searched for the website of the National Guideline Clearinghouse (https://www.ahrq.gov/gam/index.html), the International Network of Agencies for Health Technology Assessment (http://www.inahta.org/), and the Guideline International Network (https://www.g-i-n.net/) for potential unpublished guidelines. We used MeSH terms and keywords relevant to *primary aldosteronism, hyperaldosteronism, Conn's syndrome, guidelines*, and *expert consensus* to develop the search strategy (Supplementary Material 1).

Literature screen was conducted by two authors, with one author (Z.M.) acting as a clinical expert and another (C.X.) providing methodological perspectives of evidence-based practice. Titles and abstracts retrieved from the systematic literature searching were scanned, and clearly irrelevant records were excluded; full texts of remaining potentially eligible publications were obtained and assessed for a final decision based on the eligibility criteria. Any disagreements were solved through discussion by the two authors.

### The Appraisal Instrument and Quality Assessment

The Appraisal of Guidelines for Research and Evaluation (AGREE) II instrument was used for the quality assessment (14). This was an update of AGREE I by The AGREE Next Steps Consortium (15). We chose the AGREE II instrument because it has been regarded as the most comprehensive and rigorous quality assessment tool (16). The Appraisal of Guidelines for Research and Evaluation II includes 23 items structured in six domains as follows: scope and purpose (domain 1), stakeholder involvement (domain 2), rigor of development (domain 3), clarity of presentation (domain 4), applicability (domain 5), and editorial independence (domain 6) (Supplementary Material 2). Each item was rated by scores from 1 (strongly disagree) to 7 (strongly disagree) according to the extent of adherence (14). The score for each domain was derived from the obtained score (sum of score by each rater for the domain) and the maximum possible score (strongly agree) and minimum possible score (strongly disagree) (14).

The quality assessment expert group took charge of the quality assessment of included guidelines and consensuses. The group consists of two physicians (L.Z., Z.D.), three surgeons of PA (Z.M., G.Q, M.P.), and one methodologist (C.X.). Before the assessment, each group member was trained through a teleconference by the principal investigator (Z.M.) and the methodologist (C.X.) according to AGREE II user's manual. The members then assessed the quality according to AGREE II instrument independently and were required to record their decisions in a separate Excel 2010 sheet (Microsoft, Redmond, WA, USA). Except for the rater himself/herself, the results were blinded to other members.

### Data Analysis

We summarized the recommendations on the screening, diagnosis, and the treatment of each guideline and consensus. The major discrepancies among them were described. For the quality, the obtained score by each rater, maximum possible score, and minimum possible score of each domain were summarized and used to calculate the total score for each domain (17). A domain with score larger than 70% was regarded as good quality, 50 to 70% as moderate quality, and less than 50% as poor quality (17). The mean score of the six domains of each guideline was further calculated as a measurement of the overall quality of the guideline. Similarly, a guideline with the mean score of all six domains larger than 70% and the score of domains 3 and 4 larger than 70% was regarded as have good quality and could be recommended for use. We prespecified domains 3 and 4 as the most important parts because they were regarded indicative for good overall quality and a recommendation for use, respectively. The interclass correlation (ICC) was calculated for each domain, and an ICC value of 0.91 to 1.00 was regarded as excellent, 0.76 to 0.90 as good, 0.51 to 0.75 as moderate, and less than 0.50 as poor reliability (18). Data analysis was conducted using Excel 2010 software (Microsoft).

## Results

**Figure 1:**
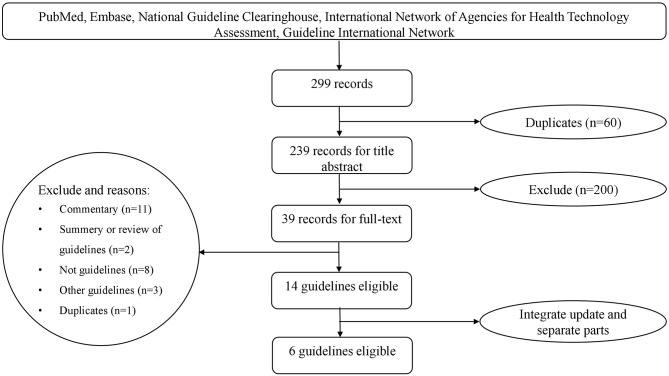
The flowchart of literature screen.

We obtained 298 records from the literature search. In addition, we obtained one guideline from the Guideline International Network (https://www.g-i-n.net/). After excluding duplicates and those that did not meet the criteria, we identified 14 potentially eligible articles (Figure 1). Of these, the 2016 Endocrine Society Clinical Practice Guideline was an update of the 2008 version; The French Endocrinology Society (SFE), in collaboration with the French Hypertension Society (SFHTA) and Francophone Endocrine Surgery Association (AFCE) consensus was divided into seven separate articles based on the topic from epidemiology to the treatment; the consensus of the Taiwan Society of Aldosteronism was divided into two separate articles, with one focused on screening and diagnosis and another focused on treatment. We finally included six guideline documents (Figure 1). Among them, three were clinical practice guidelines, and three were consensus statements. These included the Endocrine Society Clinical Practice Guideline (2016) (10), the Chinese practice consensus on the diagnosis and treatment of PA (Chinese Endocrine Society, 2016) (11), the Japan Endocrine Society guideline (2011) (12), the consensus of the Taiwan Society of Aldosteronism (2009 and 2011) (19, 20), the Clinical Management of PA by the Italian Society of Hypertension (2014) (21), and the SFE/SFHTA/AFCE consensus on PA (2016) (22–28).

### Brief Summary of the Management on PA

A brief summary of the management on PA is presented in Table 1. The baseline prevalence of PA among hypertensive patients in each guideline document ranged from 5 to 18%. Generally, current guidelines and consensuses have consistent recommendations on the diagnosis and treatment for different types of PA. All of the them recommended the use of plasma aldosterone/renin ratio (ARR) for patient screen, the computed tomography for subtype classification, the laparoscopic adrenalectomy for unilateral PA, and the mineralocorticoid receptor antagonist (spironolactone) for bilateral adrenal disease and those patients who were unable or unwilling to undergo surgery.

**Table 1:** A brief summary of the recommendations for each included guideline or consensus for primary aldosteronism (PA).

**Guidelines or consensuses**	**Prevalence of PA**	**Brief summary of the recommendation**				
		**Screen (target patients)**	**Screening test**	**Diagnosis (confirmation)**	**Diagnosis (subtype classification)**	**Treatment**
Endocrine Society 2016 (Guideline)	About 5% and possibly 10% of hypertensive patients	1. Patients with sustained blood pressure (BP) above 150/100 mm Hg on each of three measurements obtained on different days, with hypertension (BP >140/90 mm Hg) resistant to three conventional antihypertensive drugs (including a diuretic), or controlled BP (<140/90 mm Hg) on four or more antihypertensive drugs; 2. Hypertension with spontaneous or diuretic-induced hypokalemia/adrenal incidentaloma/sleep apnea/family history of early onset hypertension or cerebrovascular accident (<40 years age); and all hypertensive first-degree relatives of patients with PA.	Plasma aldosterone/renin ratio (ARR)	Patients with a positive ARR undergo one or more confirmatory tests (sodium loading test; saline infusion test; fluorohydrocortisone suppression plus oral sodium loading tests; captopril challenge test)	1. Computed tomography (CT) to determine where anatomically appropriate 2. Adrenal venous sampling (AVS) to distinct unilateral and bilateral adrenal disease 3. Genetic testing for young PA patients (<20 years) and those family history of PA or stroke at a young age (40 years)	1. Unilateral laparoscopic adrenalectomy for documented unilateral PA 2. Mineralocorticoid receptor (MR) antagonist (spironolactone) for bilateral adrenal disease and those unable or unwilling to undergo surgery 3. Low-dose glucocorticoid for glucocorticoid-remediable aldosteronism
Italian Society of Hypertension 2013 (Guideline)	More than 11% hypertensive patients	All hypertensive patients	ARR	1. No need for confirmation tests 2. Repeat the measurement of ARR (positive ARR)	1. High-resolution CT 2. AVS to distinct unilateral and bilateral adrenal disease	1. Laparoscopic adrenalectomy for patients with lateralized aldosterone secretion 2. MR antagonists for those not candidates for surgery, or show no lateralization of aldosterone secretion
Japanese Endocrine Society 2009 (Guideline)	Up to 10% in hypertensive patients	1. General practitioners: all patients initially diagnosed as hypertensive without strictly restricting blood sampling conditions 2. Specialist medical facilities: all hypertensive patients	Plasma renin activity (PRA) and plasma aldosterone concentration (PAC), and ARR (PAC/PRA) with value of >200	At least two of three confirmation tests (captopril-challenge test, upright furosemide-loading test, and saline-loading test) for patients with positive ARR	1. CT 2. AVS in candidates for surgery to determine whether aldosterone hypersecretion is bilateral or unilateral	1. Laparoscopic adrenalectomy for aldosterone hypersecretion from one adrenal 2. MR antagonists for bilateral aldosterone hypersecretion and those surgery is impossible or is not desired
Chinese Endocrine Society 2016 (Consensus)	About 7. 1% in resistant hypertensive patients	1. Sustained blood pressure >160/100 mm Hg, hypertension (>140/90 mm Hg) resistant to three conventional antihypertensive drugs, or controlled BP (<140/90 mm Hg) on four or more antihypertensive drugs 2. Hypertension with spontaneous or diuretic-induced hypokalemia/adrenal incidentaloma/sleep apnea/family history of early onset hypertension or cerebrovascular accident (<40 years age); and all hypertensive first-degree relatives of patients with PA	ARR	One or more confirmatory tests should be used (sodium loading test; saline infusion test; fluorohydrocortisone suppression plus oral sodium loading tests; captopril challenge test) for patients with positive ARR	1. CT 2. AVS to determine whether aldosterone hypersecretion is bilateral or unilateral 3. Genetic testing for young PA patients (<20 years) and those family history of PA or stroke at a young age (40 years)	1. Unilateral laparoscopic adrenalectomy for unilateral PA or aldosterone-producing adenoma 2. Mineralocorticoid receptor (MR) antagonist (spironolactone) for idiopathic hyperaldosteronism and those unable or unwilling to undergo surgery 3. Low-dose glucocorticoid for glucocorticoid-remediable aldosteronism
France SFE/SFHTA/AFCE 2016 (Consensus)	About 6–18% in patients with hypertension	1. Patients with severe hypertension (grade 3, systolic BP ≥180 mm Hg and/or diastolic BP ≥110 mm Hg) 2. Patients with resistant hypertension (≥140/90 mm Hg, despite adherence to lifestyle modifications and administration at optimal dose of ≥3 antihypertension drugs including thiazide diuretic) 3. Patients with hypertension associated with permanent or intermittent hypokalemia (<3.5 mmol/L) 4. Normal kalemia (≥3.5 to ≤ 5.0 mmol/L) but associated with another indication for PA 5. Hypertension or hypokalemia associated with an adrenal lesion of ≥10-mm diameter revealed serendipitously	ARR	One or more confirmation diagnoses should be performed for patients with positive ARR. These including intravenous saline infusion test, fludrocortisone suppression test, captopril test	1. CT (or MRI when CT is contraindicated) should be performed in all cases of PA 2. We do not recommend AVS in noncandidates for surgery 3. We suggest performing AVS in candidates for surgery aged >35 years, whatever the imaging findings. 4. Genetic testing for young PA patients (<20 years) and those family history of PA or stroke at a young age (40 years)	1. Except for adrenocortical carcinoma, the adrenal lesions causing lateralized PA are small and benign, making them ideal for laparoscopic surgery 2. When surgery is indicated, laparoscopic rather than open surgery is recommended 3. Spironolactone treatment is recommended in nonlateralized PA, and in lateralized PA for patients not wishing or unable to undergo surgery
Taiwan Society of Aldosteronism 2017 (Part I), 2019 (Part II) (Consensus)	About 16.4% in stage 3 hypertensive patients	1. Sustained systolic/diastolic blood pressure more than 150/100 mm Hg 2. Drug-resistant hypertension 3. Hypertension with spontaneous hypokalemia or diuretic-induced hypokalemia; 4. Hypertension with adrenal incidentaloma 5. Hypertension and a family history of early-onset hypertension, or cerebrovascular accident at a young age (<40 years old)6. Hypertensive patients with first-degree relatives diagnosed with PA	ARR	We suggest that one or more confirmatory tests are performed in patients with a positive ARR [saline infusion test, captopril challenge test, 24-h urine aldosterone (Uald-24 h) and random urinary aldosterone-to-creatinine ratio]	1. Genetic testing for patients with confirmed PA at age <20 years old and in those who have a family history of PA or young strokes at age <40 years old, or who still have persistent hypertension after adrenalectomy 2. Abdominal CT subgroup evaluation and to exclude large tumors suspected as adrenocortical carcinoma 3. AVS for PA patients wish to be treated surgically, to avoid unnecessary adrenalectomy	1. Laparoscopic adrenalectomy is the gold standard of care for aldosterone-producing adenoma/lateralized PA 2. In PA patients with bilateral adrenal disease or lateralized PA patients with no desire for surgical treatment, MR antagonist (spironolactone) was recommended

*The old version of Endocrine Society Clinical Practice Guideline (2008) was not appraised*.

There were several conflicts on the screen and confirmation test for PA. The Endocrine Society (2016), the Chinese Endocrine Society, the France SFE/SFHTA/AFCE, and the Taiwan Society of Aldosteronism recommended patients with high risk (e.g., sustained blood pressure) of PA should be screened (10, 11, 19, 22). However, the Italian Society of Hypertension and the Japanese Endocrine Society recommended all hypertensive patients should be screened because of the high prevalence in their country (12, 21). For the detailed target population for screen, the Endocrine Society (2016) and the Taiwan Society of Aldosteronism suggested patients with sustained blood pressure greater than 150/100 mm Hg should be screened for PA (10, 12), whereas the Chinese Endocrine Society set this cutoff point at 160/110 mm Hg, and the France SFE/SFHTA/AFCE set it as 180/110 mm Hg (11, 22). The Endocrine Society (2016) and the Chinese Endocrine Society suggested hypertensive patients with sleep apnea should be screened for PA, whereas other guidelines and consensuses did not give such a recommendation (10, 11).

There were no uniform consensuses on the detailed cutoff value of ARR as a sign for PA. Five of them recommended the confirmation test (e.g., sodium loading test; saline infusion test) for those patients with positive ARR, whereas the Italian Society of Hypertension did not recommend the use of confirmation test because these tests could lead to missing many curable cases (21). Except for the Italian Society of Hypertension and the Japanese Endocrine Society (12, 21), genetic testing was recommended for young PA patients (<20 years) and those with family history of PA or stroke at a young age (<40 years).

### Score of Each Domain of the Guidelines

Table 2 presents the score of each domain for the guidelines and consensuses according to AGREE II. There was a good reliability between the six raters (ICC ranges from 0.77 to 0.88), indicating a good agreement for the quality of our assessment. As for the most important two domains (3 and 4): for domain 3, four guideline documents have a poor quality, one has a moderate quality (Taiwan consensus), and one has a good quality (Endocrine Society practice guideline 2016); for domain 4, all of them have a good quality (score, >70%; mean score, 86.88%). All of the guideline documents have a good quality on domain 1 (mean score, 81.02%). None of the guideline documents sufficiently reported the stakeholder involvement (domain 2, mean score was 54.32%) and applicability (domain 5, mean score was 47.92%). For editorial independence (domain 6, mean score was 62.04%), only the Endocrine Society practice guideline 2016 and the France SFE/SFHTA/AFCE consensus reached a good quality.

**Table 2:** The summarized score of each domain for the PA guidelines or consensus.

**Domains**	**Endocrine Society 2016**	**Italian Society of Hypertension**	**Japanese Endocrine Society**	**Chinese Endocrine Society**	**France SFE/SFHTA/AFCE**	**Taiwan Society of Aldosteronism**	**Mean score**	**ICC**
		**Guideline**			**Consensus**			
Domain 1: Scope and purpose	91.67%	72.22%	77.78%	72.22%	91.67%	80.56%	81.02%	0.78 (0.62, 0.90)
	Good	Good	Good	Good	Good	Good	—	—
Domain 2: Stakeholder involvement	65.74%	38.89%	55.56%	46.30%	51.85%	67.59%	54.32%	0.81 (0.66, 0.91)
	Moderate	Poor	Moderate	Poor	Moderate	Moderate	—	—
Domain 3: Rigor of development	72.45%	19.73%	33.33%	43.88%	41.50%	61.90%	45.46%	0.85 (0.73, 0.93)
	Good	Poor	Poor	Poor	Poor	Moderate	—	—
Domain 4: Clarity of presentation	91.67%	76.85%	83.33%	87.04%	95.37%	87.04%	86.88%	0.88 (0.78, 0.94)
	Good	Good	Good	Good	Good	Good	—	—
Domain 5: Applicability	65.28%	28.47%	46.53%	47.22%	50.00%	50.00%	47.92%	0.77 (0.78, 0.89)
	Moderate	Poor	Poor	Poor	Moderate	Moderate	—	—
Domain 6: Editorial independence	100.00%	65.28%	40.28%	25.00%	75.00%	66.67%	62.04%	0.86 (0.74, 0.93)
	Good	Moderate	Poor	Poor	Good	Moderate	—	—
Mean score	81.13%	50.24%	56.13%	53.61%	67.56%	68.96%	—	—

*For the quality score, more than 70% is good; 50% to 70% is moderate; less than 50% is poor*.

### Quality of Each Guideline and Recommendation for Use

The mean score of each guideline and consensus across the six domains ranged from 50.24 to 81.13%. The Endocrine Society practice guideline 2016 ranked highest in overall quality, whereas the Italian Society of Hypertension ranked the lowest. For the two most important domains (3 and 4), the Endocrine Society practice guideline 2016 has a score that ranked good on quality. Based on the overall quality and score of domains 3 and 4, the Endocrine Society practice guideline 2016 was recommended for use. But it still needs some modifications especially for the stakeholder involvement and application domains. The consensus of Taiwan Society of Aldosteronism has the highest quality among the three consensuses that showed some potential for recommendation (mean score, 68.96%), whereas some improvements were needed (e.g., the rigor of development) in the further version to make it be more reliable for clinical practice. The rest, four guidelines or consensuses, referring to the quality, were suboptimal because of the unsatisfied implementation for domain 3 and/or domain 4 and the overall quality.

## Discussion

In the current report, we summarized the recommendations on the management of PA from existing guideline documents and evaluated the overall quality and the use in clinical practice. To the best of our knowledge, this is the first quality appraisal for PA guidelines. Overall, most of the recommendations by these guideline documents were consistent, although some minor conflicts existed. Our findings suggested that, based on AGREE II, for the existing guideline documents, the stakeholder involvement and applicability were insufficiently reported. Except for the Endocrine Society practice guideline 2016 (10), the development process seems to lack acceptable rigorousness. The Endocrine Society practice guideline 2016 has a good quality.

Some conflicts on the recommendations for the management of PA were observed. Several reasons may explain this. First, the prevalence of PA differs by region in that in some regions it was higher, whereas it was lower in some, which makes the recommendation on the screen different. Second, and maybe the most important one, is the lack of high-quality evidence in this area. With a brief look for the evidence used in these guidelines, we can see that the majority of which were based on the results of observational studies or expert experiences; these results were susceptible to potential bias and therefore lead to conflicting recommendations. Third, there are different medical care conditions and economic status. For example, in some regions, robot assistant surgery was used for PA, whereas in some regions it was not available for application. Fourth, the attitude for what is positive screen for PA may differ and remains debatable in this area.

In our study, we observed that the process of guideline development was suboptimal because some of them failed to employ rigorous development methods. Kent et al. (29) also reported a similar finding. As emphasized by the AGREE II tool, evidence to derive practical recommendations should be based on comprehensive literature search, clear selection criteria, and appropriate method to form the recommendations and should take both benefits and harms of interventions into consideration (16, 17). Indeed, a rigorous development process is the foundation to form trustworthy guideline recommendations, and it is the key step to build a “bridge” from high-quality evidence to the healthcare practice.

We observed that the domain of stakeholder involvement was underreported in these guidelines and consensuses. This might due to the insufficient collection of patients' views and preferences during guideline development. Similar suboptimal reporting on stakeholder involvement was documented from previous literatures (29–31). Although for physicians and surgeons, such information may have little role on the reliability of recommendation, the adoption of patients' opinions may be helpful to improve the informed decision for guideline development.

The current study conducted a critical appraisal on the guideline quality of PA based on a comprehensive literature search and a well-established instrument. Our findings may have some implications for further guidelines of PA. First, a clear description of how the evidence was searched, accessed, and linked should be clarified; moreover, a clear description on the facilitators and barriers to its application of the recommendations should be recorded; in addition, stakeholder involvement and editorial independence should be more informative.

## Conclusions

In summary, the recommendations on the management of PA were consistent among existing guidelines and consensuses, although some minor conflicts were recorded. The overall quality of the guidelines and consensuses of PA is suboptimal, and further efforts are needed to improve the quality. Taking account of overall quality and domains 3 and 4, the Endocrine Society practice guideline 2016 has the highest quality and can be recommended for use.

## Data Availability Statement

All datasets generated for this study are included in the article/Supplementary Material.

## Author Contributions

ZM, LZ, and XW proposed the ideal. ZM searched and screened the literature, and drafted the manuscript. CX screened and analyzed the data. ZM, LZ, ZD, CX, GQ, and MP assessed the quality. XW, YZ, and JK revised the manuscript.

## Conflict of Interest

The authors declare that the research was conducted in the absence of any commercial or financial relationships that could be construed as a potential conflict of interest.
