# Supplementary material for: The Quality of Clinical Practice Guidelines and Consensuses on the Management of Primary Aldosteronism: A Critical Appraisal
Source: Front Med (Lausanne). 2020 May 5;7:136. doi: 10.3389/fmed.2020.00136 (PMC7214671; doi:10.3389/fmed.2020.00136)
Supplement: Supplementary file 1 [file Table_1.DOCX]

**EMBASE**

1. Hyperaldosteronism/
2. Primary Hyperaldosteronism.ti,ab
3. Primary aldosteronism.ti,ab
4. Aldosteronism.ti,ab
5. Conn* adj1 Syndrome.ti,ab
6. Aldosterone-producing adenomas.ti,ab
7. Guidelines/
8. Practice Guideline.ti,ab.
9. Consensus.ti,ab.
10. 1 or 2 or 3 or 4 or 5 or 6
11. 7 or 8 or 9
12. 10 and 11

**PubMed**

#1 (((((Hyperaldosteronism[MeSH Terms]) OR Primary hyperaldosteronism[Title/Abstract]) OR Primary aldosteronism[Title/Abstract]) OR Aldosteronism[Title/Abstract]) OR Conn Syndrome[Title/Abstract]) OR aldosterone-producing adenomas[Title/Abstract]

#2 (((Guidelines[MeSH Terms]) OR Practice Guideline[Publication Type]) OR Guideline[Publication Type]) OR Consensus[MeSH Terms]

#3 #1 AND #2

**National Guideline Clearinghouse** (<https://www.ahrq.gov/gam/index.html>)

*No records searched*

**International Network of Agencies for Health Technology Assessment** (<http://www.inahta.org/>)

*No records searched*

**Guideline International Network** (<https://www.g-i-n.net/>)

Case detection, diagnosis, and treatment of patients with primary aldosteronism: an Endocrine Society clinical practice guideline. The Endocrine Society. NGC:006766
